# Bacterial Communities Show Algal Host (*Fucus* spp.)/Zone Differentiation Across the Stress Gradient of the Intertidal Zone

**DOI:** 10.3389/fmicb.2020.563118

**Published:** 2020-09-24

**Authors:** Charlotte T. C. Quigley, Kyle A. Capistrant-Fossa, Hilary G. Morrison, Ladd E. Johnson, Aleksey Morozov, Vicki S. Hertzberg, Susan H. Brawley

**Affiliations:** ^1^School of Marine Sciences, University of Maine, Orono, ME, United States; ^2^Josephine Bay Paul Center for Comparative Molecular Biology and Evolution, Marine Biological Laboratory, Woods Hole, MA, United States; ^3^Département de Biologie, Université Laval, Québec, QC, Canada; ^4^Center for Data Science, Nell Hodgson Woodruff School of Nursing, Emory University, Atlanta, GA, United States

**Keywords:** desiccation, *Granulosicoccus*, microbiome, *Octadecabacter*, peptide nucleic acid clamps (PNAs), Rhodobacteraceae, transplant, zonation

## Abstract

The intertidal zone often has varying levels of environmental stresses (desiccation, temperature, light) that result in highly stress-tolerant macrobiota occupying the upper zone while less tolerant species occupy the lower zone, but little comparative information is available for intertidal bacteria. Here we describe natural (unmanipulated) bacterial communities of three *Fucus* congeners (*F. spiralis*, high zone; *F. vesiculosus*, mid zone; *F. distichus*, low zone) as well as those of *F. vesiculosus* transplanted to the high zone (Dry and Watered treatments) and to the mid zone (Procedural Control) during summer in Maine (United States). We predicted that bacterial communities would be different among the differently zoned natural congeners, and that higher levels of desiccation stress in the high zone would cause bacterial communities of Dry transplants to become similar to *F. spiralis*, whereas relieving desiccation stress on Watered transplants would maintain the mid-zone *F. vesiculosus* bacterial community. Bacteria were identified as amplicon sequence variants (ASVs) after sequencing the V4 hypervariable region of the 16S rRNA gene. Microbiome composition and structure were significantly different between the differently zoned congeners at each tissue type (holdfasts, receptacles, vegetative tips). ASVs significantly associated with the mid-zone congener were frequently also present on the high-zone or low-zone congener, whereas overlap in ASVs between the high-zone and low-zone congeners was rare. Only 7 of 6,320 total ASVs were shared among tissues over all congeners and transplant treatments. Holdfast bacterial community composition of Dry transplants was not significantly different from that of *F. spiralis*, but Watered holdfast communities were significantly different from those of *F. spiralis* and not significantly different from those of procedural controls. Additional stressor(s) appeared important, because bacterial communities of Dry and Watered transplants were only marginally different from each other (*p* = 0.059). The relative abundance of Rhodobacteraceae associated with holdfasts generally correlated with environmental stress with highest abundance associated with *F. spiralis* and the two high-zone transplant treatments. These findings suggest that the abiotic stressors that shape distributional patterns of host species also affect their bacterial communities.

## Introduction

Intertidal zones are dynamic ecosystems where multiple abiotic stresses (e.g., temperature, desiccation, light, nutrient) and biotic interactions (e.g., competition, facilitation, parasitism, predation) occur with differing intensities across a few vertical meters. Often, abiotic stresses determine the upper distributional limit for macrobiota (e.g., Baker, 1909, 1910; Doty, 1946; Schonbeck and Norton, 1978, 1979; Dethier et al., 2005), whereas biotic interactions determine the lower limit (e.g., Connell, 1961a, b; Paine, 1974; Lubchenco, 1980; Hartnoll and Hawkins, 1985). This is not an absolute rule, however (e.g., Underwood, 1980; Hartnoll and Hawkins, 1985). As diverse research continues to deepen our understanding of the distributions of intertidal macrobiota (e.g., see references in Benedetti-Cecchi and Trussell, 2014; Hurd et al., 2014; Monteiro et al., 2019), it is equally important to investigate how intertidal bacterial communities are distributed, what influences their distribution, and how these bacterial communities influence other organisms, including the macroalgae that often dominate intertidal communities.

Bacteria can affect macroalgae in diverse ways – both positively and negatively. Some symbiotic bacteria are vital to their host because they produce morphogens that induce and maintain normal algal morphology (e.g., Provasoli and Pintner, 1980; Matsuo et al., 2005; Spoerner et al., 2012; Tapia et al., 2016; Ghaderiardakani et al., 2017), reduce the amount of biofouling on the algal surface (Holmström et al., 1996; Rao et al., 2007; Nasrolahi et al., 2012; Singh and Reddy, 2014), protect against pathogens (Longford et al., 2019; Saha and Weinberger, 2019) or produce essential vitamins or siderophores (Helliwell et al., 2011; Dogs et al., 2017; Karimi et al., 2020). For example, Fries (1977) demonstrated experimentally that some bacteria are required for normal development in *Fucus spiralis*, because only callus developed when antibiotics were used to remove bacteria. Furthermore, many bacteria belonging to the phyla Bacteriodetes, Proteobacteria, Planctomycetes, and Verrucomicrobia produce cellulases, alginases, and/or fucosidases (Thomas et al., 2012; Kim et al., 2016; Sichert et al., 2020) that allow them to derive carbon from digestion of brown algal cell walls (Ivanova et al., 2002), but, at the same time, some produce morphogens, vitamins, etc. for their algal hosts (e.g., Ghaderiardakani et al., 2017). Finally, bacteria can be opportunistic pathogens, possibly due to abiotic or biotic stresses decoupling mutualistic relationships (Egan et al., 2014; Kumar et al., 2016).

Bacterial biodiversity often varies among different macroalgal hosts in a single location (Lachnit et al., 2009; Weigel and Pfister, 2019), within the same macroalgal host in different biogeographic regions (Roth-Schulze et al., 2018; Morelan et al., 2019), by tissue (Staufenberger et al., 2008; Quigley et al., 2018; Weigel and Pfister, 2019; Paix et al., 2020), or due to abiotic changes (Tujula et al., 2010; Michelou et al., 2013; Miranda et al., 2013; Longford et al., 2019; Qiu et al., 2019; Saha et al., 2020). Extensive studies of the biodiversity of bacteria on Baltic Sea *Fucus vesiculosus*, which is not usually exposed to air (Medvedev et al., 2016), found effects of different salinities (Stratil et al., 2014) and higher temperature (Stratil et al., 2013; Mensch et al., 2016). Experimental work with surface extracts from Baltic Sea *Fucus vesiculosus* suggests that this alga and/or its epiphytes may actively attract or repel some bacteria (Lachnit et al., 2010, 2013; Wahl et al., 2010; Saha et al., 2011, 2012, 2014; Parrot et al., 2019). While bacteria from the surrounding environment undoubtedly colonize macroalgae, parental effects on bacterial biodiversity are also possible. For example, the egg-containing oogonia of *F. vesiculosus* appear to be colonized from the parental thallus surface as they are released into the sea (Goecke et al., 2012).

Here, we focus on bacterial communities associated with a model intertidal system of three fucoid congeners that are distributed across the vertical stress gradient associated with aerial (i.e., tidal) exposure: *Fucus spiralis* L. (high zone), *Fucus vesiculosus* L. (mid zone), and *Fucus distichus* subsp. *edentatus* Powell (low zone). These species are all canopy-forming bioengineers that have similar morphologies and share many traits; however, they have different statures (height of *F. spiralis* < *F. vesiculosus* < *F. distichus*), and some physiological and biochemical differences (e.g., cell wall fucoidan content, scavenging of reactive oxygen stress, heat shock proteins; Baker, 1910; Dring, 1982; Mabeau and Kloareg, 1987; Collén and Davison, 1999a, b; Collén and Davison, 2001; Li and Brawley, 2004) cause fundamentally different tolerances to environmental stresses that correlate with their positions in the intertidal zone. Bacterial communities on *Fucus* congeners may contribute also to differences in host stress tolerance because studies across phylogenetically diverse taxa found that bacteria appear to aid their host’s tolerance to thermal (Dunbar et al., 2007; Xie et al., 2013; Ziegler et al., 2017) or drought/salinity stresses (Zolla et al., 2013; Rolli et al., 2015; Dittami et al., 2016; Yuan et al., 2016).

Little is known about the microbial communities associated with *Fucus* spp. in the North Atlantic intertidal zone. In particular, we do not know if bacteria exhibit classic patterns of vertical zonation, and, if they do, whether such patterns are caused by abiotic stresses (e.g., desiccation) or biotic interactions (e.g., the distribution of host species, changes in host physiology). To further knowledge in this area, we had five main objectives: (1) determine how bacterial communities of *Fucus* spp. vary among the three different species; (2) determine how the bacterial communities of *Fucus* spp. vary by tissue type (i.e., holdfasts, receptacles (reproductive tissue), vegetative tips); (3) determine if the bacterial communities of the *Fucus* host differ from the seawater-associated bacteria; (4) determine if the specific bacterial community of a given fucoid species can be altered by imposition of different levels of environmental stress on the host congener; and (5) determine if the effect of abiotic stress can be ameliorated by reducing desiccation stress. To achieve these objectives, we combined a natural survey of fucoid bacterial communities across the low, mid, and high intertidal zones with a novel transplant experiment of the mid-zone species (*F. vesiculosus*) to the high zone where stresses associated with aerial exposure are higher.

## Materials and Methods

### Field Site and Design

**FIGURE 1:**
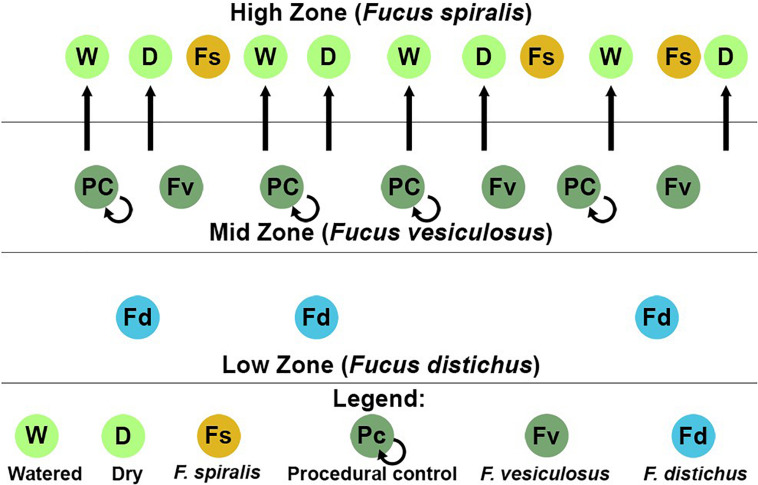
Sampling scheme for both the natural survey and transplant experiment in one of the two study areas at Schoodic Point, Maine (United States). Dry (D), procedural control (PC), and watered (W) refer to our transplant treatments for *Fucus vesiculosus*.

**FIGURE 2:**
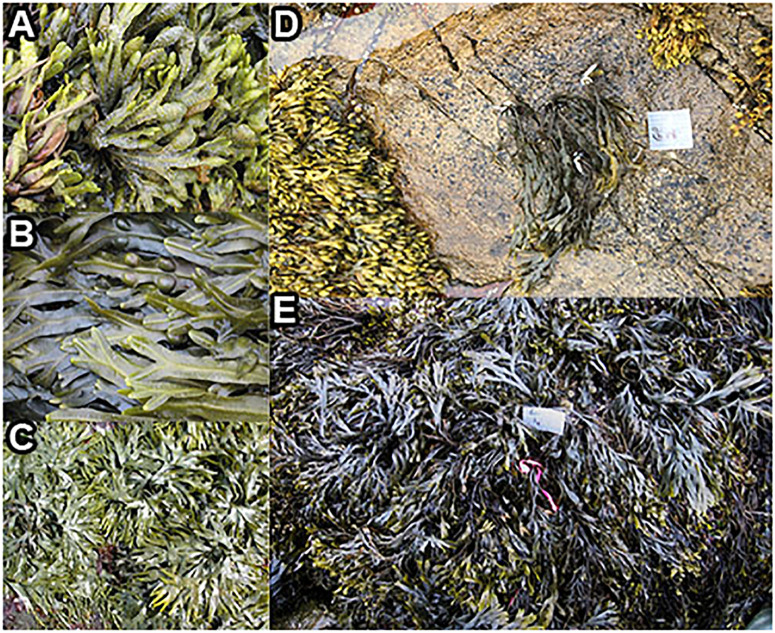
*Fucus* congeners, and transplanted *F. vesiculosus* from Schoodic Point during summer 2016. Natural communities of **(A)** *F. spiralis*, **(B)** *F. vesiculosus*, and **(C)** *F. distichus*; **(D)** an example of a clump of *F. vesiculosus* that was transplanted from the mid zone to the high zone; **(E)** a clump of *F. vesiculosus* (near pink tag) transplanted into the mid zone as a procedural control.

We conducted a simultaneous natural survey and transplant experiment (Figure 1) of high zone *F. spiralis* (Figure 2A), mid zone *F. vesiculosus* (Figure 2B) and low zone *F. distichus* subsp. *edentatus* (Figure 2C) to study the bacterial communities of differently zoned *Fucus* congeners at Schoodic Point (Acadia National Park, ME, United States; National Park Service permit: ACAD-2016-SCI-0010). Bacterial samples from natural (unmanipulated) congeners were collected on 6 July 2016 and 20 July 2016. Samples from *F. vesiculosus* back-transplanted to the mid zone as a procedural control or transplanted to the high zone in two different treatments (see below) were also sampled on these 2 days, along with an intermediate timepoint (11 July 2016). The transplant study was limited to 2 weeks to avoid possible damage to transplanted individuals (Baker, 1909; Schonbeck and Norton, 1978). We used two study areas: Area A (44.333085, −68.058391) and Area B (44.333952, −68.058106), and established a 30-m transect through the middle of each congener’s distribution in each area by marking the ends with stainless steel screws to create high-zone, mid-zone, and low-zone transects. Transects for the transplant experiment (in each area’s high and mid zones) were close to the natural transects but were laid to avoid overlap.

### Variation in Bacterial Communities Among Tissues, Congeners, and the Water Column

During the natural surveys, we removed the holdfast, several vegetative blade tips, and several receptacles (the reproductive organ found on some blade tips) from randomly selected individuals of each species (*n* = 3 individuals/transect) at randomly selected 1-m intervals along each transect, using sterile techniques (Figure 1). Each tissue sample was placed in a sterile, 50-mL plastic Falcon tube containing 35 mL of sterile natural seawater (salinity = 32) and shaken up and down vigorously for 10 s to remove loosely attached (transient) bacteria. Natural seawater for rinses was sourced from the University of Maine’s coastal Center for Cooperative Aquaculture Research (CCAR), and was UV sterilized and 1-μm filtered at CCAR. The seawater was then autoclaved and stored at 4°C until use. Rinsed samples were drained of seawater, transported on ice (4°C), flash-frozen ∼6 h later in liquid nitrogen, and stored at −80°C. Because each species was differently zoned, we predicted differences in bacterial communities among congeners (i.e., **Hypothesis 1**). Furthermore, we predicted that the different functions supported by each tissue would cause significant differences among the bacterial communities at the level of tissue (i.e., **Hypothesis 2**).

Water samples (1 L) were collected above each transect before ebb tide uncovered the macroalgae on each sampling date. After transport to the lab on ice (4°C), water samples were pre-filtered through a 5.0-μm sterile filter and finally filtered through a 0.2-μm sterile filter to recover the bacterial community of the water columns. Filters were then flash-frozen and stored at −80°C. We predicted that the bacteria of all sampled tissues from unmanipulated *Fucus* congeners and experimental transplants of *F. vesiculosus* would differ from bacteria in the water column (i.e., **Hypothesis 3**).

### Transplant Experiments: Manipulations of Stress

On 6 July 2016, we transplanted *F. vesiculosus* from the mid zone to the high zone in the proximity of (but not touching) *F. spiralis* and as back-transplanted procedural controls (PC) within the mid zone (Figure 1). To do so, intact individuals were collected within the mid zone of each area using gloves, immediately and randomly assigned to clumps of 4 individuals, and fastened to the rock individually using eye bolt anchors and zip ties to mimic an algal canopy, with holdfasts on the substratum (Figure 2D). We created 8 experimental clumps in each of the two transects in the high zone as well as 4 clumps as procedural controls along transects in each mid-zone area (Figure 2E). During the next 2 weeks, four of the clumps in each of the high-zone transects were kept wet by pouring seawater over them every 30 min (Watered treatment, W) during the time that the mid zone was underwater during the daytime low tide. The remaining clumps were allowed to experience high intertidal stresses without any alleviation (Dry treatment, D). Treatments were spatially interspersed to avoid pseudoreplication. Seawater for watering was collected from accessible points over the *F. vesiculosus* zone as the tide fell or rose and several liters of seawater were poured over each clump until it and the underlying substratum were wet. A different team member tended each sampling area, to ensure the treatment was applied at each interval over ∼ 5 min. Vegetative blade tips were harvested from a different individual in each clump on different sampling days to avoid inducing defensive responses to wounding; several receptacles, vegetative tips, and the holdfast were collected from the central individual in each clump on 20 July 2016. We did not attempt to transplant *F. vesiculosus* to the low zone, because the greater number of biotic interactions would have confounded a field transplant experiment. We predicted that the bacterial communities of *F. vesiculosus* transplanted to the high zone would become different over time from bacterial communities of natural and procedural controls of *F. vesiculosus* in the mid zone (i.e., **Hypothesis 4**). More specifically, we predicted that desiccation would be the major stress causing this transition, therefore the bacterial community of W would remain similar to mid-zone communities, whereas D would develop communities similar to *F. spiralis* (i.e., **Hypothesis 5).**

### Sample and Bioinformatic Processing

For each individual, a vegetative tip, a receptacle, and the holdfast were separately pulverized using sterile technique, and 25 mg dry wt of each sample transferred to a microfuge tube. Samples were powdered using a Geno/Grinder (SPEX SamplePrep, Metuchen, NJ; 2 min, 1600 strokes/min, 2.4-mm zirconium beads). DNAs were extracted from each powdered sample using a Qiagen DNeasy Plant MiniKit (Germantown, MD, United States) according to the manufacturer’s protocol. We analyzed whole samples (rather than swabs) of tissues, because not all macroalgal bacteria are epiphytic. Consequently, the V4 hypervariable region of the 16S rRNA gene was amplified in the presence of peptide nucleic acid clamps (PNAs, 25 μM) that we developed to block the amplification of host 18S rRNA and plastid genes (Supplementary Table S1). The amplification primers contained Illumina-specific sequences for binding to the flow cell and sequencing primers fused to 16S GT-515F (GT-GTGYCAGCMGCCGCGGTAA) and CC-806RB (CCGGACTACNVGGGTWTCTAAT). The forward primer contained an in-line barcode, and the reverse primer contained an index captured by a short indexing read. Cycling conditions were 94°C for an initial denaturation of 3 min, 30 cycles of 94°C for 45 s, 78°C for 10 s, 50°C for 1 min and 72°C for 90 s and a final extension at 72°C for 10 min. The products were cleaned, quantified, and pooled as previously described (Quigley et al., 2018). Amplicon pools (up to 96 amplicon libraries) were sequenced on an Illumina MiSeq (manufacturer’s protocol, v.3 sequencing kit). Because of low or no amplification, 14 of the 240 samples were excluded from analysis (see Supplementary Table S2 for final sample sizes).

Paired-end reads were demultiplexed by index using on-instrument software and by barcode using a custom python script (Quigley et al., 2018). Paired-end reads were merged, trimmed of primer sequences, and quality filtered (Eren et al., 2013). The final datasets served as input to Minimum Entropy Decomposition (MED) analysis (Eren et al., 2015). The MED analysis returned 17,553,478 high-quality, merged V4 reads. The average number of reads across the 226 samples was 77,670 (±48,693 [SD]), ranging from 13,578 to 485,446 reads. We created a reference V4 16S rDNA database from the SILVA reference taxonomy v.132^1^ (Pruesse et al., 2007). The MED analysis identified 6,320 ASVs (amplicon sequence variants) that had ≥ 10 assigned reads across all sequenced samples. We subsampled without replacement (rarefied) to an even sampling depth of 17,000 high-quality, merged sequences/sample. Taxonomy was assigned to these ASVs using VSEARCH (Rognes et al., 2016) and our custom V4 database. To improve taxonomic resolution, assignments with poor identification (e.g., “uncultured bacterium”) were amended by searching the NCBI non-redundant nucleotide BLAST database in addition to the SILVA reference, examining the taxonomic distribution of the matches, and creating a placeholder taxonomy based on the highest taxonomic level (e.g., “Proteobacteria_cl_or_fam_gen” would represent an unknown genus within the Proteobacteria). We refer to ASVs by concatenating their assigned ASV number to their genus-level taxonomy (e.g., *Granulosicoccus*_3260). Raw fastq files of sequences can be found at the NCBI Sequence Read Archive, accession number PRJNA481673.

### Statistical Analysis

#### *A Priori* Hypothesis Testing

All statistical analyses were performed using R v. 3.6.1 (R Core Team, 2019). Code used for performing each analysis can be found at www.github.com/kacf24/FucusTransplant. To evaluate our hypotheses based on community composition and structure we used subsets of our data to create multivariate generalized linear models (MGLMs; Wang et al., 2012). Models based on community **composition** were created by transforming abundance data into a presence/absence matrix and assuming a binomial distribution for the model, whereas community **structure** models used untransformed abundance data and a negative-binomial distribution (Roth-Schulze et al., 2018). Separate models were created to examine differences in bacterial communities between (1) species and tissues in unmanipulated (natural) *Fucus* spp.; (2) algal species, transplant treatments, and the surrounding water column; (3) sampling date and transplant treatment for vegetative tissues; and (4) treatments and tissues among *F. vesiculosus* transplants and unmanipulated *F. vesiculosus* and *F. spiralis*. The fit of each model was checked by looking for the absence of a strong pattern in residual plots (Wang et al., 2012), and the significance of each model was tested through ANOVA testing (anova.manyglm function). Factors with *p* < 0.05 were considered significant. Significant interaction terms were further analyzed in each model through pairwise testing (pairwise.comp argument of anova.manyglm function). Finally, to understand which individual ASVs had significantly different composition or structure among different natural congeners or experimental treatments, we used univariate testing and adjusted for multiple comparisons (p.uni = “adjusted” in anova.manyglm function). To identify tissues, species, or treatments with which these significantly different ASVs were associated, we then applied indicator species analysis (De Cáceres and Legendre, 2009) to reveal ASVs significantly indicative of different conditions within our tested models. Indicator species analysis determines these significant associations based on an ASV’s group fidelity and specificity (De Cáceres and Legendre, 2009). Fidelity is the probability of finding the ASV in a given group, and specificity is the probability of finding the ASV in a group given its presence (De Cáceres and Legendre, 2009). To visualize community structure differences for each model, we created an analogous NMDS plot using appropriate subsets of data and Bray-Curtis dissimilarity (metaMDS function, R package vegan; Oksanen et al., 2019). The stress levels (a measure of how well the 2D image represents the data) were recorded.

#### Description of Communities

Core communities were created for each of the three tissues (“Holdfast,” “Receptacle,” and “Vegetative”) within each species or transplant treatment. This study defines core communities as ASVs that were present in ≥50% of replicate samples collected from holdfasts, receptacles, or vegetative tips among species or treatment at the end of the experiment (i.e., 20 July 2016).

Bar charts showing information at the taxonomic level of class were created by pooling together ASVs of a given class by all replicates of a given sample type (treatment/species) and tissue. Then, the proportion of each class in the tissue (per treatment or species) pool was calculated. Only classes accounting for >170 (i.e., 1% of reads in a sample) reads were included. Family level information was generated similarly in a scatterplot, except that means instead of proportions were calculated by adding together all reads of a given family and dividing by the number of replicates among tissue/species or treatment. Only families with a mean > 170 reads/sample were displayed. Data were plotted using ggplot2 (Wickham, 2016). Additionally, the 10 most abundant ASVs in each tissue of every congener and transplant treatment were tabulated.

#### Verification of Relative Environmental Stress Levels Between the High and Mid Zones

Although the high intertidal zone usually has higher levels of environmental stress than the mid zone, we verified this in the two intertidal areas used for our studies. Thermal loggers (iButton, DS1921G-F5#, Maxim, San Jose, CA, United States) measured microhabitat temperatures from 24 June 2016 to 15 July 2016 every 15 min. Epoxy putty (Z-Spar Splash Zone Epoxy [A-788], West Marine, Watsonville, CA, United States) was used to encapsulate each parafilm-wrapped sensor and attach it to the substratum while providing thermal characteristics similar to the rock. The sensors were deployed as pairs with one logger under fucoid canopy (covered, C) while the second was just outside that area of canopy (exposed, E). Eight pairs were deployed over the mid zone or high zone (*n* = 2 pairs/transect/zone). A programming error led to a premature end in data collection for one exposed iButton in the upper intertidal zone. We used a LI-COR spherical quantum sensor (SPQA3718, Lincoln, NE, United States) connected to a LI-COR light meter (LI-250, Lincoln, NE, United States) to measure irradiance every 30 min during low tide from 10 July to 18 July 2016.

iButton records were linked to the tidal height recorded at a nearby NOAA buoy (#8413320, Bar Harbor, ME, United States), and time series using these data were then filtered to contain only temperatures of daytime exposures to compute descriptive statistics. Hierarchical clustering visualized similarities between iButtons based on Euclidean dissimilarity (pairwise differences) of Z-Score normalized iButton records. Records were then clustered using Ward’s sum of square errors method (Suzuki and Shimodaira, 2006; Murtagh and Legendre, 2014) to verify expected levels of relative stress between the mid zone and high zone.

## Results

### Specificity of Bacterial Communities to Congeners and Tissues (Hypotheses 1, 2)

**FIGURE 3:**
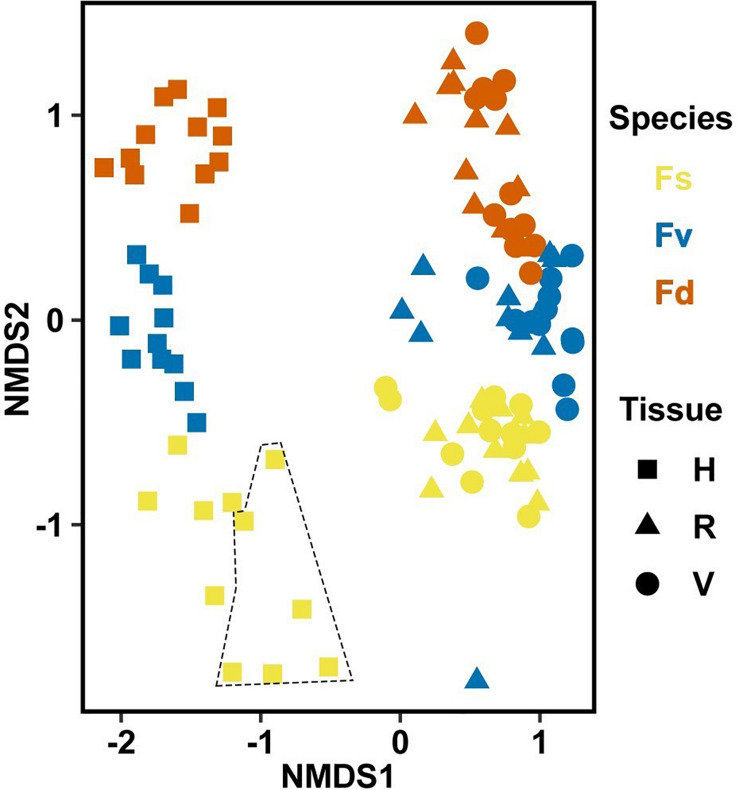
Non-metric multidimensional scaling (NMDS) using Bray–Curtis dissimilarity for all samples of natural congeners (*n* = 105). Fs, *Fucus spiralis*; Fv, *Fucus vesiculosus*; Fd, *Fucus distichus*; H, holdfast; V, vegetative; and R, receptacles. All samples within the encircled area are from study Area A. Stress = 0.106.

Significantly different bacterial communities were found among the differently zoned *Fucus* species (*p* = 0.001; Table 1A), and among tissues (*p* = 0.001; Table 1A), but with a significant species:tissue interaction (*p* = 0.001, Table 1A). Pairwise tests of the interaction revealed that bacterial composition and structure were significantly different (*p* = 0.001, Supplementary Figure S1) between species at each tissue type. No within-species test of composition or structure between receptacles and vegetative tips was statistically significant (*p* > 0.07, structure; *p* > 0.121, composition), except for the structure test for *Fucus distichus* (*p* = 0.029; Supplementary Figure S1). Despite the unpredicted lack of differentiation of receptacle and vegetative bacteria within some species (Figure 3 and Supplementary Figure S1), we found strong differentiation of bacterial communities in the differently zoned congeners across all tissues, with holdfast communities being distinct from vegetative and receptacle tissues both within and across congeners (Figure 3). The greater variability of replicate points in the NMDS for *Fucus spiralis* holdfasts was associated with differences in the high-zone transects between study areas (Figure 3).

**TABLE 1:** Results of analysis of deviance tests comparing composition and structure of MGLMs (Wang et al., 2012) of **(A)** Species and tissues of *Fucus* congeners; **(B)** Algal species, transplant treatments, and the surrounding water column; **(C)** Sampling date and transplant treatment for vegetative tissues; and **(D)** Treatments and tissues among *F. vesiculosus* transplants and unmanipulated *F. vesiculosus* and *F. spiralis*.

(A) *Fucus spiralis, Fucus vesiculosus*, and *Fucus distichus*							

**Data subset: All Fs, Fv, and Fd (*n* = 105)**							

Composition				Structure			
Factor	Levels	Deviance	*P*-value	Factor	Levels	Deviance	*P*-value
Day	July 6, July 20	5211.39	0.176	Day	July 6, July 20	5675.72	0.067
Area	A, B	5446.52	0.118	Area	A, B	5807.21	0.058
Species	Fs, Fv, Fd	27223.86	0.001*	Species	Fs, Fv, Fd	29588.67	0.001*
Tissue	H, R, V	39907.85	0.001*	Tissue	H, R, V	43233.12	0.001*
Species:Tissue		4571.27	0.001*	Species:Tissue		7379.93	0.001*

**(B)** Procedural control, Dry, Watered, *Fucus spiralis*, *Fucus vesiculosus*, *Fucus distichus*, and Water Column							

**Data subset: All samples (*n* = 226)**							

**Composition**				**Structure**			
**Factor**	**Levels**	**Deviance**	***P*-value**	**Factor**	**Levels**	**Deviance**	***P*-value**

Sample Type	Fs, Fv, Fd, D, W, PC, WC	65366	0.001*	Sample Type	Fs, Fv, Fd, D, W, PC, WC	99092.73	0.001*

**(C) Procedural Control, Dry, and Watered Vegetative Tissue**							

**Data subset: All vegetative tissue from PC, D, W (*n* = 68)**							

**Composition**				**Structure**			
**Factor**	**Levels**	**Deviance**	***P*-value**	**Factor**	**Levels**	**Deviance**	***P*-value**

Area	A, B	2899.08	0.032*	Area	A, B	3114.11	0.022*
Day	July 6, July 11, July 20	5152.34	0.001*	Day	July 6, July 11, July 20	5233.73	0.001*
Treatment	D, W, PC	5796.93	0.001*	Treatment	D, W, PC	6127.12	0.001*
Day:Treatment		2208.88	0.001*	Day:Treatment		3023.34	0.001*

**(D) Procedural control, Dry, Watered, *Fucus spiralis*, and *Fucus vesiculosus***							

**Data subset: All PC, D, W, Fs, Fv from 20 July 2016 (*n* = 101)**							

**Composition**				**Structure**			
**Factor**	**Levels**	**Deviance**	***P*-value**	**Factor**	**Levels**	**Deviance**	***P*-value**

Area	A, B	5283.31	0.08	Area	A, B	5770.32	0.016*
Tissue	H, R, V	38840.86	0.001*	Tissue	H, R, V	40751.88	0.001*
Treatment	D, W, PC, Fv, Fs	27775.39	0.001*	Treatment	D, W, PC, Fv, Fs	29800.7	0.001*
Tissue:Treatment		6585.26	0.004*	Tissue:Treatment		10161.35	0.001*

Deviance is the result of likelihood ratio tests and factors with higher values are more explanatory for the model. We consider p < 0.05 as significant (*).

### Congener/Zone-Specific ASVs

**FIGURE 4:**
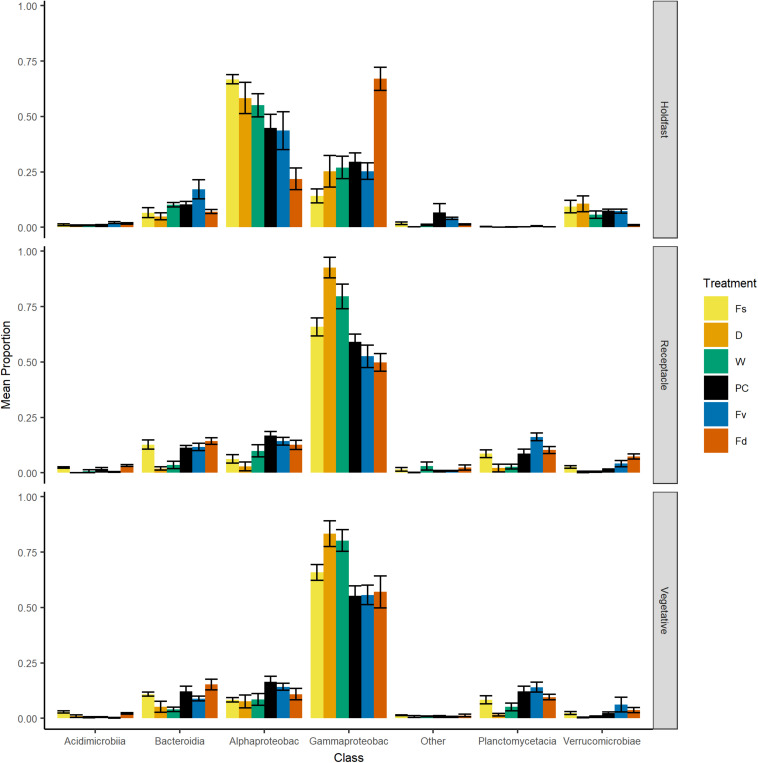
Bar plot of relative proportion (± SE) of major classes of bacteria across replicates within: Fs, *Fucus spiralis*; D, dry transplant of *Fucus vesiculosus*; W, watered transplant of *Fucus vesiculosus*; PC, procedural control of *Fucus vesiculosus*; Fv, natural *Fucus vesiculosus*; and Fd, *Fucus distichus*. Class abbreviations: Alphaproteobac, Alphaproteobacteria and Gammaproteobac, Gammaproteobacteria.

Over 2000 ASVs belonging to 19 phyla were present on each congener (*F. spiralis* = 2046 ASVs, *F. vesiculosus* = 2422 ASVs, *F. distichus* = 2644 ASVs, Supplementary Table S3). Most of these ASVs belonged to the Proteobacteria, which on average contained over half the reads in our natural congeners (Figure 4). Alphaproteobacteria were more relatively abundant for holdfasts of *F. spiralis* and *F. vesiculosus*, whereas Gammaproteobacteria were more relatively abundant for holdfasts of *F. distichus*. Gammaproteobacteria were also more relatively abundant on vegetative tips and receptacles across all congeners than any other class (Figure 4). Planctomycetacia were more relatively abundant than Verrucomicrobiae on vegetative tips and receptacles of congeners, but Verrucomicrobiae were more abundant on holdfasts than Planctomycetacia. The relative abundance of Acidimicrobiia and Bacteroidia varied among congeners (Figure 4).

**FIGURE 5:**
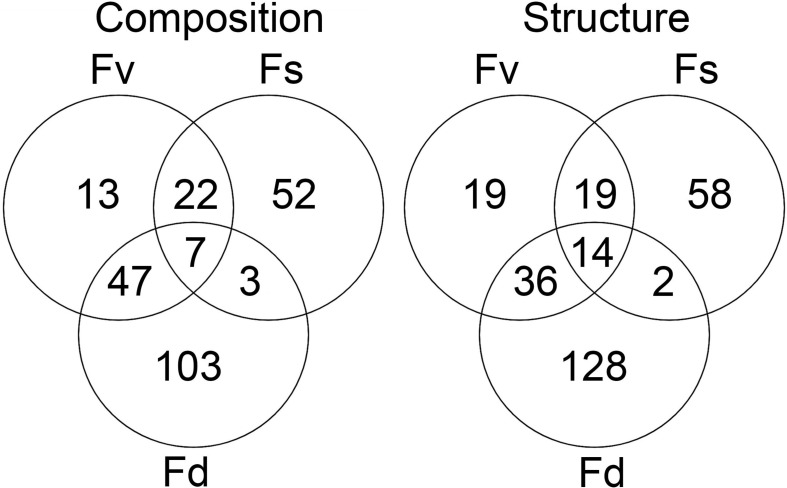
The number of significantly different ASVs (*p* < 0.05 in mvabund analysis) identified as being indicative of *Fucus spiralis*, *Fucus vesiculosus*, or *Fucus distichus*.

Many ASVs had significantly different (*p* < 0.05, mvabund) distributions among the *Fucus* congeners (Supplementary Table S4). Using indicator species analysis, only a small group of these ASVs were significantly associated across the intertidal stress gradient with all three congeners (composition: 7 ASVs, structure: 14 ASVs; Figure 5). The high-zone *F. spiralis* and the low-zone *F. distichus* had few indicator species in common (Figure 5; composition: 3 ASVs, structure: 2 ASVs). In contrast, many indicator ASVs were shared between *F. spiralis* and the mid-zone *F. vesiculosus* (Figure 5; composition: 29 ASVs, structure: 33 ASVs) and between *F. distichus* and *F. vesiculosus* (Figure 5; composition: 54 ASVs, structure: 50 ASVs).

ASVs in some genera (e.g., *Granulosicoccus*, *Octadecabacter*) occurred disproportionately across tissues among congeners in core communities (Table 2). For example, *Octadecabacter* was more common in holdfast cores than in receptacle or vegetative cores. Within holdfasts there were 15 unique *Octadecabacter* ASVs associated with *F. spiralis* (high zone); 15 unique ASVs associated with *F. vesiculosus* (mid zone), and 2 unique ASVs associated with *F. distichus* (low zone). Four unique ASVs were shared among all three species, and in addition, *F. spiralis* and *F*. *vesiculosus* shared 4 unique ASVs, and *F. vesiculosus* and *F. distichus* 6 unique ASVs; however, no ASVs were shared only between high-zone *F. spiralis* and low-zone *F. distichus*. Comparable differences in distribution were found for ASVs within other genera (Table 2).

**TABLE 2:** Distribution of the number of core ASVs among selected genera across the holdfast, receptacle, and vegetative core communities of *Fucus spiralis* (Fs), *Fucus vesiculosus* (Fv), and *Fucus distichus* (Fd).

Tissue	ASV Genus	Fs + Fv + Fd	Fs	Fs + Fv	Fv	Fv + Fd	Fd	Fd + Fs	Total Fs	Total Fv	Total Fd
Holdfast	Total # of ASVs	38	97	34	161	112	144	8	177	345	302
	*Granulosicoccus*	6	3	1	4	5	8	0	10	16	19
	*Octadecabacter*	4	15	4	15	6	2	0	23	29	12
	*Burkholderia-Caballeronia-Paraburkholderia*	1	1	0	1	2	5	1	3	4	9
	*Sulfitobacter*	2	5	1	1	0	2	1	9	4	5

Receptacle	Total # of ASVs	66	77	20	44	47	143	13	176	177	269
	*Granulosicoccus*	20	14	3	9	6	17	0	37	38	43
	Octadecabacter	2	1	0	1	0	2	1	4	3	5
	*Burkholderia-Caballeronia-Paraburkholderia*	2	5	0	0	0	0	0	7	2	2
	*Sulfitobacter*	2	0	0	0	0	0	1	3	2	3

Vegetative	Total # of ASVs	68	84	26	53	47	96	8	186	194	219
	*Granulosicoccus*	24	11	6	13	5	8	1	42	48	38
	Octadecabacter	1	1	1	3	0	0	0	3	5	1
	*Burkholderia-Caballeronia-Paraburkholderia*	2	5	0	0	0	0	3	10	2	5
	*Sulfitobacter*	1	1	2	0	0	0	0	4	3	1

### Comparison of Bacterial Communities in the Water Column and *Fucus* spp. (Hypothesis 3)

**FIGURE 6:**
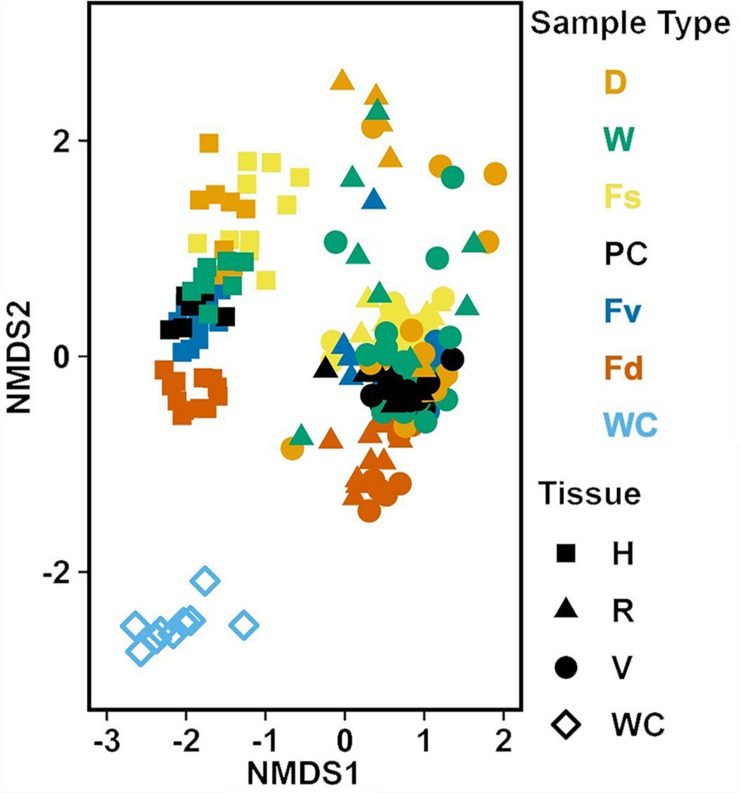
Non-metric multidimensional scaling (NMDS) using Bray–Curtis dissimilarity for all samples (*n* = 226). D, dry transplant; W, watered transplant; Fs, *Fucus spiralis*; PC, Procedural control; Fv, *Fucus vesiculosus*; Fd, *Fucus distichus*; WC, water column; H, holdfast; R, receptacles; and V, vegetative. Stress = 0.142.

Analysis of the MGLMs of all samples (Table 1B) revealed a significant difference in composition and structure based on the source of the sample (i.e., water column, congener, or transplant treatment). Pairwise testing then revealed that the water column was significantly different (all pairwise *p* < 0.014) from each algal sample type (Figure 6). The water column possessed many unique taxonomic groups at all levels (Supplementary Table S3) and lacked taxonomic groups common to all algal samples (e.g., Melainabacteria). Furthermore, many of the families that were relatively abundant in the water column are not so in any algal samples (Supplementary Figure S2).

### Response of Bacterial Communities to Transplant of Mid-Zone *F. vesiculosus* to the High Zone

#### Changes in Bacterial Communities on Vegetative Tissue Over Time (Hypothesis 4)

**FIGURE 7:**
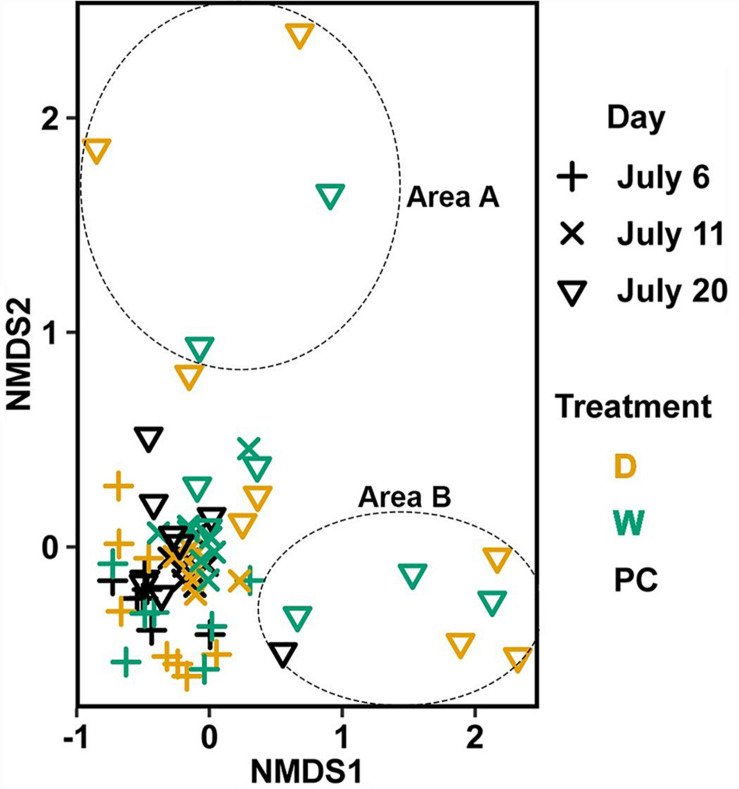
Non-metric multidimensional scaling (NMDS) using Bray–Curtis dissimilarity for vegetative tissue from transplant treatments (*n* = 68). D, dry transplant; W, watered transplant; Fs, *Fucus spiralis*; PC, Procedural control; Fv, *Fucus vesiculosus*; Fd, *Fucus distichus*; H, holdfast; R, receptacles; and V, vegetative. Stress = 0.141. The encircled samples from July 20 are driving spread in the plot and are from different sampling areas, but some samples from both areas (not circled) overlapped.

Significant differences (Table 1C) in bacterial communities on vegetative tips over the three sampling dates were found to be related to sample area, treatment (PC, W, D), and the duration (day) of treatment (Hypothesis 4), along with a day:treatment interaction. However, pairwise testing comparisons were largely non-significant for both composition and structure (Supplementary Figure S3). The only significant comparison was between D on 20 July and PC on 6 July (Supplementary Figure S3). Inspection of the NMDS (Figure 7) shows a higher degree of variability in datasets from July 20 compared to July 6. There appear to be two distinct groups of datasets that are driving this variability and are a mixture of both D and W samples, but the groups come from the different sample areas (Figure 7).

#### Zone/Congener Position and Stress Level Influence on Bacterial Community Structure (Hypothesis 5)

**FIGURE 8:**
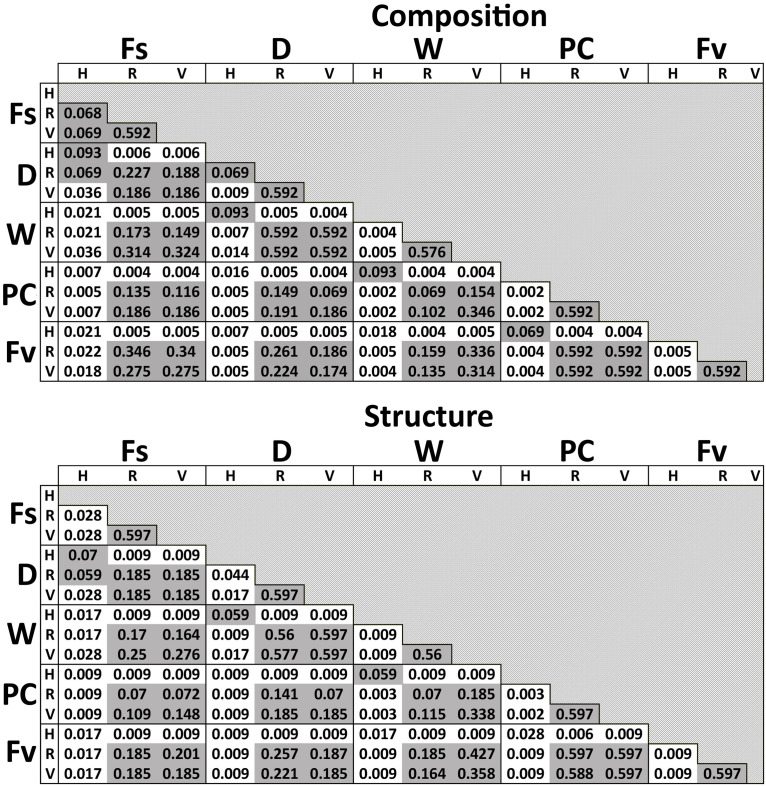
Adjusted *p*-values of pairwise comparisons of microbial composition and structure for the significant interaction term (Tissue:Treatment) over the three transplant treatments (Dry, Watered, Procedural control), unmanipulated *Fucus vesiculosus* and unmanipulated *Fucus spiralis*. Shaded values are not significant (*p* > 0.05).

**FIGURE 9:**
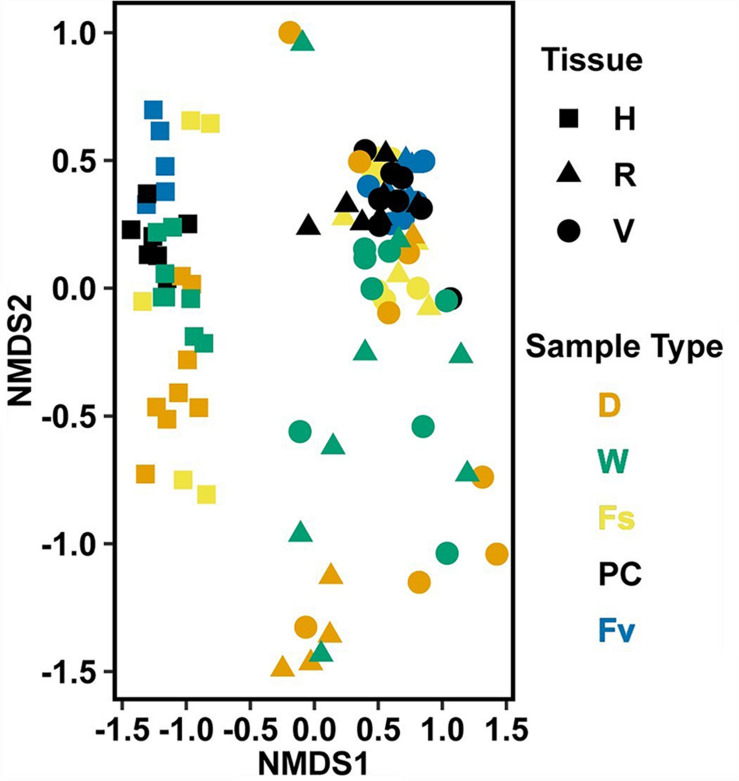
Non-metric multidimensional scaling (NMDS) using Bray–Curtis dissimilarity for transplant treatments, *Fucus vesiculosus*, and *Fucus spiralis* on 20 July 2016 (*n* = 101). Figure legend as in Figure 6. Stress = 0.150.

Significant differences in bacterial communities were found across the transplant experiment and natural *F. vesiculosus* and *F. spiralis* related to tissue, treatment and, for structure analyses, area (Table 1D). Analysis of the significant tissue:treatment interactions (Table 1D) in pairwise tests (Figure 8) found that bacterial communities of the holdfast had changed as predicted (Hypothesis 5). Bacterial communities of holdfasts were not significantly different between the D transplant treatment and natural *F. spiralis* in either composition or structure pairwise tests, whereas both were significantly different from PC and Fv. The prediction (Hypothesis 5) that W (seawatered treatment) would not be significantly different from the PC treatment was supported by pairwise comparisons of bacterial composition and structure (Figure 8). Thus, for the holdfast, relief of desiccation stress appeared to maintain the *F. vesiculosus* mid-zone community, whereas D treatments changed toward the high-zone *F. spiralis*. PC holdfast communities became significantly different (*p* = 0.028) from natural Fv in structure analyses but not composition analyses (*p* = 0.069). Pairwise comparisons of D versus W in structure (*p* = 0.059) and composition analyses (*p* = 0.093) were not significantly different, which suggests that both high-zone transplants were experiencing stresses in addition to desiccation stress in the high zone. There were no significant differences between the receptacle and vegetative communities (composition or structure) of any group (Figures 8, 9). The mean relative proportions of different classes across natural congeners and particular transplant treatments were generally consistent with the ASV-level statistical results, especially for holdfast Alphaproteobacteria (Figure 4).

#### Identification of Indicator ASVs

Univariate testing identified many ASVs that were significantly different among the five groups tested in the transplant experiment [composition: 126 ASVs, structure: 117 ASVs (Supplementary Table S5)]. Nearly a third of these ASVs (composition: 39 ASVs, structure: 48 ASVs) were indicative of a single group, and *F. spiralis* and dry transplants were the two groups with the largest number of indicator ASVs (Supplementary Table S5). Despite this, the phylum-level diversity was highest in *F. spiralis* > PC = *F. vesiculosus* > Watered transplants > Dry transplants (Supplementary Table S5). No Cyanobacteria were identified as significant indicators in these tests. Some ASVs (composition: 13 ASVs, structure: 10 ASVs) were shared indicators for both watered and dry high-zone transplants, and 3 ASVs in the structure analysis were indicative of all high-zone groups (i.e., *F. spiralis*, dry transplant and watered transplant). In comparison, many ASVs (composition: 35 ASVs, structure: 28 ASVs) were indicators of the mid-zone groups only (i.e., Procedural Control and natural *F. vesiculosus*). A few ASVs (composition: 4 ASVs, structure: 2 ASVs) were indicative of all *F. vesiculosus* (natural, D, PC, W). Overall, in both composition and structure tests, the two most common genera that held indicator ASVs were *Granulosicoccus* and *Psychromonas* (Supplementary Table S5).

#### Core Community Analyses

Seven ASVs (Supplementary Table S6) were shared among ≥ 50% of transplant treatment replicates (i.e., the transplant core from dry, procedural control, and watered treatments) but were absent in ≥50% of unmanipulated congener replicates (“natural core”). In contrast, only 1 ASV was found in all congener cores without being present in one or more transplant cores (Supplementary Table S6). Seven ASVs were present in ≥50% of replicates in all transplant and natural congener communities, regardless of tissue. Cores of the same tissue had many shared ASVs regardless of transplant treatment or species (holdfast: 30 ASVs, receptacle: 28 ASVs, vegetative: 39 ASVs). Within core communities, ASVs within the same genus had differential distributions across tissues, congeners, and/or treatments (Table 3). For example, ASVs belonging to *Blastopirellula*, *Granulosicoccus*, *Octadecabacter*, *Roseibacillus*, and *Sulfitobacter* were present in all groups, but while receptacle/vegetative richness > holdfast richness in most groups, richness of *Octadecabacter* and *Sulfitobacter* was higher in holdfast communities (Table 3). In contrast, some genera (e.g., *Maribacter*) were present evenly across cores (Table 3). Other genera (e.g., *Pleurocapsa*, *Marinicella*) were only found in either receptacle/vegetative or holdfast cores (Table 3). Likewise, some ASVs were present in all groups except one (e.g., *Leucothrix*, absent from most *F. spiralis* cores), present in a single core (e.g., *Euzebya* and *Rubrivirga* only in *F. spiralis* cores; Table 3), or present in a treatment group (e.g., *Psychromonas* in all transplant treatments). Overall, richness in holdfast cores > receptacle cores > vegetative cores (Table 3).

**TABLE 3:** Differential distributions of ASVs within genera among different core community groups.

Genus	Holdfast						Reproductive						Vegetative					
	*Fucus spiralis*	Dry	Watered	Procedural Control	*Fucus vesicu losus*	*Fucus distichus*	*Fucus spiralis*	Dry	Watered	Procedural Control	*Fucus vesicu losus*	*Fucus distichus*	*Fucus spiralis*	Dry	Watered	Procedural Control	*Fucus vesicu losus*	*Fucus distichus*
*Blastopirellula*	2	1	2	2	4	4	10	6	6	13	19	21	9	7	8	17	19	19
*Euzebya*	1	0	0	0	0	0	1	0	0	0	0	0	1	0	0	0	0	0
*Maribacter*	3	3	4	4	3	4	2	1	7	4	4	2	2	1	5	3	5	2
*Rubrivirga*	1	0	0	0	0	0	1	0	0	0	0	0	2	0	0	0	0	0
*Winogradskyella*	2	6	11	9	4	2	1	0	0	0	0	0	1	0	0	0	0	0
*Pleurocapsa*	0	0	0	0	0	0	1	1	2	3	2	4	1	2	2	2	2	2
*Granulosicoccus*	11	6	10	11	16	19	38	6	16	38	38	43	42	17	21	37	48	38
*Leucothrix*	0	1	3	3	1	5	1	1	1	7	11	24	0	3	1	7	6	13
*Litorimonas*	2	3	5	3	0	1	4	3	5	8	9	9	5	4	5	9	16	12
*Marinicella*	1	2	2	4	5	5	0	0	0	0	0	0	0	0	0	0	0	0
*Octadecabacter*	23	36	43	33	29	12	4	2	6	7	3	5	3	4	7	7	5	1
*Psychromonas*	0	14	5	5	0	0	0	36	3	3	0	0	0	6	2	2	0	0
*Sulfitobacter*	9	6	7	6	4	5	2	2	3	3	3	2	4	2	4	3	3	1
*Roseibacillus*	1	1	4	4	6	3	6	1	3	10	7	15	5	3	3	10	8	14

#### Highly Abundant Taxa

**FIGURE 10:**
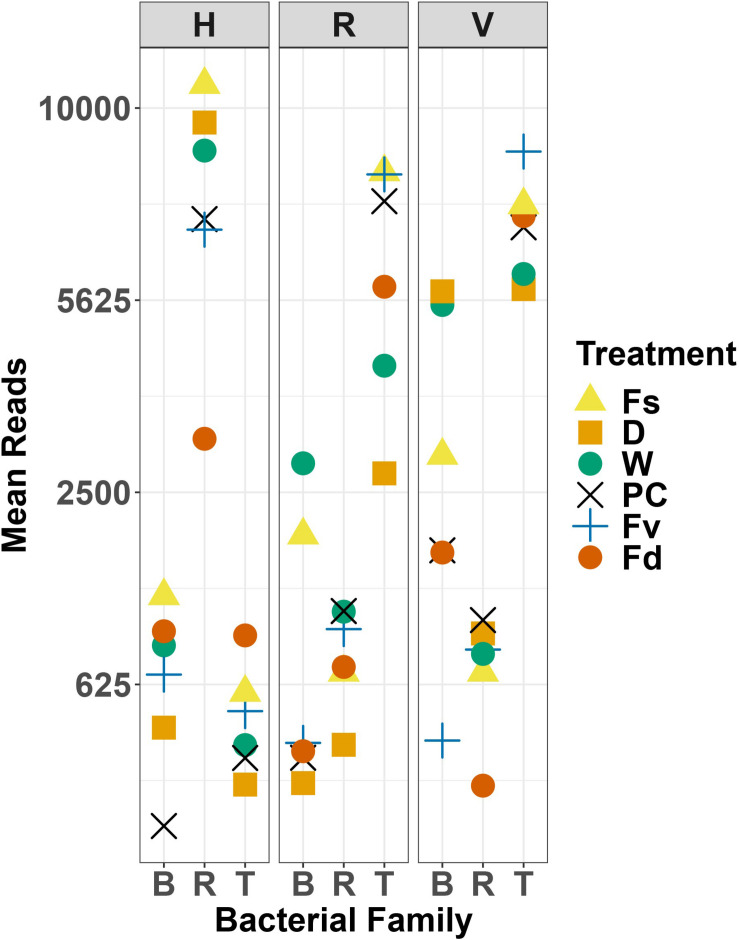
Scatter plot of mean reads of selected major families of bacteria across replicates. D, dry transplant; W, watered transplant; Fs, *Fucus spiralis*; D, dry transplant; W, watered transplant; PC, procedural control, Fv, *Fucus vesiculosus*; Fd, *Fucus distichus*; H, holdfast; R, receptacles; V, vegetative; B, Burkholderaceae; R, Rhodobacteraceae; and T, Thiohalorhabdaceae.

Holdfast samples were dominated by Alphaproteobacteria (highest in Fs) and Gammaproteobacteria (highest in Fd; Figure 4). Additionally, holdfast samples had a relatively even abundance of Bacteriodia and Verrucomicrobiae, except for a low abundance of Verrucomicrobiae in *F. distichus*. Receptacles and vegetative tips were dominated by Gammaproteobacteria, but the relative abundance was highest in high-zone samples (Fs, D, W; Figure 4). Verrucomicrobiae were nearly absent from the high-zone transplants, but Alphaproteobacteria, Bacteroidia, and Planctomycetacia were present in receptacles and vegetative tissues of all samples (Figure 4). Prominent families from natural or experimental fucoid tissues included Rhodobacteraceae (Alphaprotobacteria); Flavobacteriaceae and Saprospiraceae (Bacteriodetes); Burkholderiaceae, Pseudoalteromonadaceae, Psychromonadaceae, and Thiohalorhabdaceae (Gamma proteobacteria); and the Pirellulaceae (Planctomycetacia) (Figure 10 and Supplementary Figure S2). The abundance of Rhodobacteraceae in holdfast communities (Figure 10) was higher in groups based on their natural or transplanted zone (i.e., degree of abiotic stress) with a significant effect of group (one-way ANOVA, *F*_5,33_ = 6.01; *p* = 0.0005). *Post hoc* comparison of groups (Tukey HSD test) revealed three overlapping sets of groups: a (Fs, D, and W), ab (PC and Fv), and b (Fd). Relatively few genera composed the ten most abundant ASVs across congener and transplant tissues, and they were distinct from the most abundant genera in the water column (Supplementary Table S7). The most abundant ASVs in receptacles and vegetative tips tended to be shared among the natural congeners and the procedural control. *Granulosicoccus*_3260 and *BurkholderiaCaballeroniaParaburkholderia*_8371 were the two most abundant ASVs (Supplementary Table S7).

### Environmental Analysis to Compare Abiotic Stress Levels Between Zones

Higher levels of abiotic stress occurred in the high zone compared to the mid zone during our study. Temperatures were up to 10°C higher for the exposed (outside canopy) versus covered (underneath canopy) iButtons of adjacent pairs in either the high zone (*F. spiralis*) or mid zone (*F. vesiculosus*) (Supplementary Table S8). Mid-zone maximum temperatures were 4–6°C lower than maximum temperatures in the high zone (e.g., canopy-covered iButtons between high zone and mid zone at site A, Supplementary Table S8). The mean daily exposure at low tide was 2–4 h longer in the high zone compared to the mid zone (Supplementary Table S8) although due to topographic differences between transects, the length of aerial exposure varied between the two high-zone transects. Mean temperatures and ranges in temperature at iButton sites between the high and mid zones offered little differentiation when compared to total exposure periods and temperature maxima to explain any stress-related differences in bacterial communities found associated with *Fucus* spp. A spring/neap tidal cycle coincided with our experiment, which decreased exposure times near the midpoint (11 July 2016) of the experiment. Hierarchical clustering based on all time points (i.e., low tide and high tide, day and night) found that thermal sensors clustered by their position in the intertidal zone, with 100% approximately unbiased *p*-values and 100% bootstrap probabilities (Supplementary Figure S4), showing that zone was the main factor accounting for differences in temperature records. Additionally, records clustered differently between exposed and covered iButtons (highly supported; ≥ 98%, Supplementary Figure S4).

Mean morning (<11:00 am), midday (11:00 am – 1:00 pm), and afternoon (>1:00 pm) LI-COR irradiance measurements were 1918 ± 811 μmol photons/m^2^/s (*n* = 34), 2350 ± 585 μmol photons/m^2^/s (*n* = 32), and 1674 ± 678 μmol photons/m^2^/s (*n* = 30), respectively. Algae and bacterial communities experienced daytime irradiances between 142 and 2815 μmol photons/m^2^/s when exposed to the air.

## Discussion

This study supports our general hypothesis that bacteria have different distributions across the vertical stress gradient of the intertidal zone, similar to intertidal macrobiota, through these findings: (1) congener-indicative ASVs were most abundant on *F. spiralis* and *F. distichus*, which shared few ASVs; (2) only a few ASVs were found across all congeners and tissues; (3) only a few ASVs were highly abundant on all congeners and transplants (particularly *Granulosicoccus*_3260 and *BurkholderiaCaballeroniaParaburkholderia*_8371); (4) bacterial communities of holdfasts of dry transplants of *F. vesiculosus* were not significantly different from *F. spiralis*, whereas communities on watered transplants were not significantly different from procedural controls of *F. vesiculosus*; (5) the relative abundance of Rhodobacteraceae among natural and transplanted congeners at the end of the experiment correlated to the environmental stress levels of each zone and treatment; and (6) within ASV-rich genera (e.g., *Granulosicoccus*, *Octadecabacter*), many ASVs were unique to a particular congener. This last finding offers a potential parallel to the differential stress tolerance of the host *Fucus* spp. that results in these species occupying different zones in the North Atlantic intertidal zone and shows again the power of ASV definition to provide ecological insights (Eren et al., 2015).

The two types of statistical comparisons (Supplementary Figure S1 and Figure 8) involving the bacterial communities of natural *F. spiralis* and natural *F. vesiculosus* gave different results for differences in receptacle and vegetative bacterial communities between these two congeners. In the natural congener test (Supplementary Figure S1), each species’ receptacle or vegetative bacterial communities were significantly different from every other species; whereas, when bacterial communities of the three transplant treatments were included (Figure 8), the significant species:tissue interaction did not find statistically significant differences between receptacle bacterial communities or vegetative bacterial communities between natural *F. spiralis* and *F. vesiculosus*. We attribute this difference to the lower degree of power in the transplant analysis (Figure 8), which had 5 main comparisons (vs. 3 for the congener analysis) and only used data from the July 20 sampling of the natural congeners to make the sample sizes comparable with those of the transplants. Similar studies in the future should accordingly increase sample sizes, which might lead to additional interesting results for changes in bacterial communities of vegetative tips and receptacles in the transplant experiment, similar to those documented here for the holdfasts.

Through our transplant experiment, we attempted to determine if observed bacterial responses were due to differential intertidal stresses *per se* while recognizing that they may also be influenced by characteristics of the host, host responses to stress, or even the presence of other organisms (e.g., grazers, epiphytic diatoms). We formed clumps of *F. vesiculosus* individuals in the high zone to simulate canopy conditions under dry or watered treatments for 2 weeks, because earlier work found less stress-resistant *Fucus* species died over time when exposed experimentally to the air (Baker, 1909) or transplanted into higher zones (Schonbeck and Norton, 1978) for longer periods. *Fucus vesiculosus* transplants to the high zone clearly experienced more environmental stress than back-transplanted controls, because their time out of water (or “emersion”) at low tide was 2–4 h longer and the maximal environmental temperatures were 4–6°C higher. When judged at a macroscopic level (i.e., visual appearance of host individuals over time), our manipulation to relieve desiccation on watered versus dry treatments appeared successful during the experimental period, because watered transplants retained the color and texture of mid-zone *F. vesiculosus*. Within 1–2 weeks after our experiment ended, thalli in the dry treatment had reddish-brown areas and subsequently became so brittle that wave breakage ensued. Watered transplants went through similar changes over time, albeit more slowly, and some thalli were still partially intact until early October.

Our transplant of the mid-zone species to the high zone did not have a rapid effect on the bacterial communities, and differences only appeared after 14 days (D and W vs. PC treatments). However, while watering transplants relieved desiccation, which we hypothesized would influence bacterial responses, a consistent result was that the composition and structure of bacterial communities between watered and dry holdfasts, receptacles, or vegetative tips were not significantly different. Because desiccation relief probably also lessened thermal stress, we hypothesize that longer exposure to the high levels of light (visible and UV, see below) experienced during emersion could be a stronger stress on bacterial communities in the high zone than previously recognized. Different intertidal bacteria have photoprotective pigments (e.g., carotenoids, melanins), or photoactivated ion pumps (rhodopsins); additionally, some have light harvesting pigments (bacteriochlorophyll, Dogs et al., 2017) that make them subject to reactive oxygen stress under high light levels (Tomasch et al., 2011). Thus, greater exposure to light may also be driving the intertidal stress gradient for bacteria. Testing this hypothesis would, however, be difficult in the field, because it would require spectrally fitted, open-sided awnings to discriminate the relative effects of light and desiccation on bacteria of dry versus watered transplants, because even mesh awnings reduce temperature and desiccation (Williams et al., 2013). Mesocosms and laboratory experiments may thus be necessary to determine the underlying physical basis of this interesting result. They would also allow for greater control and monitoring of epiphytic organisms, which will have their own bacterial communities and may confound field results. An alternative and complementary approach would be to conduct laboratory experiments with isolates of ASVs that belong to the same genus (e.g., *Granulosicoccus*, *Octadecabacter*) but were differently zoned among *Fucus* congeners. Subjecting such isolates to different levels of stresses (e.g., temperature, desiccation, light [visible, UV]) would help determine whether the differential distributions of ASVs we report here are due to bacterial responses to differential intertidal stresses *per se* or may also be influenced by host responses to stress or incidental to other organisms (e.g., grazers, epiphytic diatoms). Furthermore, some of the differentially distributed ASVs we discovered could be used to test mechanisms affecting intertidal microbial ecology, just as *Fucus* spp. have been used for over a century.

The power of novel experimental approaches is well illustrated by the elegant experiments of Lachnit et al. (2010, 2013), who deployed gels underwater that were perfused continuously with surface extracts of *Fucus* that were prepared from submerged Baltic Sea *F. vesiculosus*. Lachnit et al. (2010) showed that delivery of isolated fractions of natural, surface-associated compounds from *Fucus* resulted in recruitment of epibacteria on the gel surface within 7 days that was similar to those observed on the natural *Fucus*. For example, perfused non-polar fractions containing fucoxanthin and other unidentified compounds reduced the abundance of epiphytic bacteria on *F. vesiculosus*, while polar fractions attracted Vibrionales (Lachnit et al., 2013). Fucoxanthin, a photosynthetic pigment and antioxidant in stramenopile algae (Graham et al., 2016; Wells et al., 2017) such as *Fucus*, was found on the surface of *F. vesiculosus* at ecologically relevant concentrations by Saha et al. (2011). A perfused gel would be difficult to use (without confounding influences) in the intertidal zone, but it would be interesting to develop a durable artificial substratum (see Brawley and Johnson, 1991) that could be initially established with biofilms of conspecific ASVs (or coated with *Fucus* extract) along the intertidal stress gradient to compare recovered ASVs to those on congener *Fucus*. The role of surface attractants in influencing the bacterial communities of intertidal *Fucus* is important to consider, especially because it may have a graded effect due to moister conditions at low tide in the low intertidal zone while stressful levels of desiccation retard fouling by epiphytes on macroalgae in the high intertidal zone (e.g., reviewed for commercial application to aquaculture by Blouin et al., 2011). It is clear that there are many biotic interactions among epiphytic bacteria, fungi, diatoms, and other microscopic algae that influence the recovered bacterial communities (and composition of surface extracts) from *Fucus* (e.g., Saha et al., 2012; Parrot et al., 2019). One surface-associated compound of *Fucus vesiculosus*, dimethylsulfoniopropionate (DMSP), acts as an antimicrobial compound (Saha et al., 2012) but the surface-associated DMSP may be produced by epiphytes [algae (Broadbent et al., 2002) or bacteria (Curson et al., 2017)] of *Fucus*. Established axenic model systems such as the green alga *Ulva compressa* “mutabilis” show unequivocally how important release of DMSP is to attracting morphogenetic bacteria such as the alphaproteobacterium *Roseovarius* to a callus of *Ul*va, which leads to normal algal development, while the bacterium uses glycerol released by *Ulva* as a source of carbon for bacterial growth (Kessler et al., 2018). Whether DMSP is made by *Fucus* or its epiphytes, it clearly has an effect on the holobiont’s microbial community. For example, *Granulosicoccus* and *Octadecabacter* both possess genes for DMSP degradation (Kang et al., 2018; Zeng and Qiao, 2019), and they account for 10% of all ASVs in our study. These genes may contribute to the presence of these genera on many invertebrates and macroalgae (e.g., Miranda et al., 2013; Roth-Schulze et al., 2018; Lemay et al., 2018; van de Water et al., 2018; Weigel and Pfister, 2019).

Another long-recognized biotic effect on the biodiversity of macroalgal epibacteria and other epiphytes is the remarkable ability of many macroalgae to slough off the outer layer of their thick cell walls, causing bacteria/epiphytes to be lost as the cell wall peels off [e.g., green algae: intertidal *Ulva intestinalis* (Figure 10 in McArthur and Moss, 1977); brown algae: rockpool *Halidrys siliquosa* (Figures 6, 9 in Moss, 1982), intertidal *Himanthalia elongata* (Figures 1, 2 in Moss, 1982), intertidal *Ascophyllum nodosum* (Figures 2, 5 in Halat et al., 2020; also see Filion-Myklebust and Norton, 1981)]. Halat et al. (2015) estimated that the abundant, mid-intertidal fucoid *Ascophyllum nodosum* sheds 25% of its epidermis every week. *Ascophyllum* is not common on shores as exposed as our field site, but on moderately exposed shores of the North Atlantic intertidal zone, it often co-occurs with *F. vesiculosus*. This is clearly an exciting time of converging investigations that may lead to elucidation of the interplay of abiotic and biotic factors that shape the macroalgal microbial community (and see Wichard and Beemelmanns, 2018).

Intertidal bacteria living on macroalgae across the vertical stress gradient of the intertidal zone might be expected to have some of the same stress-tolerant mechanisms that their hosts have, but different degrees of stress tolerance depending upon bacterial position in the intertidal zone. Many environmental stresses cause a build-up of reactive oxygen species (ROS), so mechanisms to detoxify ROS are needed (Davison and Pearson, 1996). For example, *F. spiralis* produces higher amounts of ROS-scrubbing enzymes than *F. distichus* (Collén and Davison, 1999a). It is likely that intertidal macroalgae receive further protection from ROS damage through mutualisms with bacteria that are catalase-rich (Morris et al., 2011). Overall, high-zone species of macroalgae possess greater ROS metabolism, as well as broader thermal (Schonbeck and Norton, 1978; Li and Brawley, 2004) and light (Altamirano et al., 2003) tolerances, and these traits would likely be present in high-zone bacteria. For example, known species of *Kytococcus*, a genus of Actinobacteria associated with *F. spiralis* in this study, can be pigmented, produce catalase, and have an optimum growth rate at temperatures similar to maxima recorded in this study (i.e., 28–37°C; Whitman, 2015). Likewise, known species of *Rubrivirga*, a genus in the thermophilic Rhodothermaceae here associated with *F. spiralis*, may produce catalase and oxidase, possess a suite of rhodopsins, and can resist a broad range of temperatures (4–42°C; Whitman, 2015). The only described *Schleiferia* species was isolated from a hot spring (Albuquerque et al., 2011); possibly, the *Schleiferia* ASVs found in this study in the high zone may have similar tolerances. Some Rhodobacteraceae are associated with thermally stressed corals (Pootakham et al., 2019), their abundance was positively correlated with the incubation temperature of *F. vesiculosus* cultures (Stratil et al., 2013), and they were the most abundant family found on *F. spiralis* (Dogs et al., 2017). Here, the abundance of Rhodobacteraceae in holdfast communities was highest in the high zone and generally correlated with levels of intertidal stress. Overall, such thermally tolerant bacteria appear to possess mechanisms that would allow them to survive in the upper intertidal zone, but consequently they may lack the ability to compete with bacteria typical of lower zones. Low-zone algae are typically stronger competitors than higher-zoned, more stress-tolerant species, and if bacteria fit into similar models, many of the ASVs associated with *F. distichus* may have traits such as higher rates of division and good motility, which would allow them to dominate space, as in the competitive exclusion of other species by *Chondrus crispus* in the low zone (Lubchenco, 1980). Additionally, low-zone bacteria might produce antimicrobial compounds, allowing them to gain and hold space against other competitors [e.g., other bacteria; similarly to interference competition by production of phlorotannin and galactolipids in *F. vesiculosus* (Deal et al., 2003; Brock et al., 2007)] and/or exhibit niche partitioning [e.g., to avoid direct competition for the same resources such as seen for bryozoans on *Fucus serratus* (O’Connor et al., 1980)]. ASVs of *Aquimarina*, which were present in the *F. distichus* holdfast core community, are resistant to many antibiotics (benzylpenicillin, carbenicillin, gentamicin, kanamycin, neomycin, polymyxin B, streptomycin, and tetracycline), can produce antibiotics, and possess the capability to digest multiple algal polysaccharides (Whitman, 2015; Keller-Costa et al., 2016; Hudson et al., 2019). Similarly, ASVs belonging to *Litorimonas* were associated with *F. distichus*, and members of this genus are resistant to a wide variety of antibiotics [e.g., polymyxin B, streptomycin, gentamicin, kanamycin, novobiocin, oleandomycin, lincomycin, ampicillin, tetracycline, penicillin G, carbenicillin (Jung et al., 2011; Schellenberg et al., 2018)]. These groups demonstrate that low-zone bacteria do possess some of the expected traits of low-intertidal organisms and would be expected to outcompete high-zone bacteria. This hypothesis is testable by isolating ASVs from different zones and conducting competition experiments in the laboratory.

Some bacterial associates of *Fucus* spp. may contribute to their host’s resilience to abiotic and biotic stresses across the intertidal stress gradient. We would expect bacteria that are known to produce morphogens and vitamins to be among such associates, as well as bacteria that produce antibiotic compounds that might control pathogens. We recovered ASVs belonging to genera that affect the growth rate and/or support normal development in brown, green or red macroalgae in previous studies, including *Halomonas* (Tapia et al., 2016; Ghaderiardakani et al., 2017), *Maribacter* (Ghaderiardakani et al., 2017), *Sulfitobacter* (Amin et al., 2015; Grueneberg et al., 2016), and *Roseobacter* (Tapia et al., 2016). During stressful conditions, macroalgae may need increased levels of morphogens, and many ASVs identified in the study belonged to morphogen-producing genera. Finally, some of the recovered bacteria belong to known vitamin B_12_-producing bacteria including *Loktanella*, *Octadecabacter*, and *Sulfitobacter* (Dogs et al., 2017), which were present across all tissues, species, and treatments in our study, hypothetically making vitamin B_12_ available to their algal hosts. Vitamin B_12_ (cobalamin) is an essential vitamin for fucoid algae (Fries, 1993), and these genera may contribute to algal growth during thermal stress in the high zone. Indeed, vitamin B_12_-producing bacteria aid the unicellular freshwater alga *Chlamydomonas reinhardtii* during thermal stress (Xie et al., 2013).

We identified seven universally present ASVs (i.e., ASVs present in all core communities): *Blastopirellula*_628, *BurkholderiaCaballeroniaParaburkholderia*_8371, two *Granulosi-coccus* (ASVs 3260 and 3316), *Ilumatobacter*_6062, *Octadecabacter*_12254, and *Roseibacillus*_10361. These ASVs compose a universal core microbial group for *Fucus* spp. at least during the summer at Schoodic Point. Given their distribution across multiple zones, these ASVs are likely strong competitors and stress tolerant. Although some of these ASVs (*Granulosicoccus*_3316, *Roseibacillus*_10361) were universally present, they also had significantly different distributions between species or treatments in mvabund tests. This variation suggests that although these species are commonly present, their abundances are responding to some biological or physical environmental factor.

Differently zoned species of *Fucus* have long been an important model system for descriptive and experimental studies of the abiotic and biotic interactions that shape community structure in the intertidal zone. Here we have extended such studies to the microbial communities associated with these intertidal foundation species. We found very few bacterial ASVs that were distributed widely across host congeners; instead, different ASVs, even within the same bacterial genus, were often significantly associated with one congener or with two congeners in bordering zones, or with a particular treatment with different stress levels in our transplants. Targeted isolations of such ASVs can now be followed with experimental work in the laboratory and mesocosms to define whether they have fundamentally different levels of tolerance to environmental stresses.

## Data Availability Statement

The datasets presented in this study can be found in online repositories. The names of the repository/repositories and accession number(s) can be found below: https://www.ncbi.nlm.nih.gov/, accession number PRJNA481673.

## Author Contributions

SB, HM, LJ, and CQ planned the research. CQ, KC-F, LJ, and SB conducted the field work. CQ, AM, and HM conducted the DNA extractions, sequencing, and bioinformatic analyses. KC-F performed the statistical analysis. KC-F, SB, CQ, HM, LJ, AM, and VH wrote the manuscript and approved submission of the final manuscript. All authors contributed to the article and approved the submitted version.

## Conflict of Interest

The authors declare that the research was conducted in the absence of any commercial or financial relationships that could be construed as a potential conflict of interest.

## Footnotes

1 https://www.arb-silva.de
