# Supplementary material for: Bacterial Communities Show Algal Host (Fucus spp.)/Zone Differentiation Across the Stress Gradient of the Intertidal Zone
Source: Front Microbiol. 2020 Sep 24;11:563118. doi: 10.3389/fmicb.2020.563118 (PMC7541829; doi:10.3389/fmicb.2020.563118)
Supplement: Supplementary file 1 [file Image_1.pdf]

Composition

		Fs			Fv			Fd		
		H	R	V	H	R	V	H	R	V
Fs	H									
	R	0.001								
	V	0.001	0.153							
Fv	H	0.001	0.001	0.001						
	R	0.001	0.001	0.001	0.001					
	V	0.001	0.001	0.001	0.001	0.121				
Fd	H	0.001	0.001	0.001	0.001	0.001	0.001			
	R	0.001	0.001	0.001	0.001	0.001	0.001	0.001		
	V	0.001	0.001	0.001	0.001	0.001	0.001	0.001	0.121	

Structure

		Fs			Fv			Fd		
		H	R	V	H	R	V	H	R	V
Fs	H									
	R	0.001								
	V	0.001	0.123							
Fv	H	0.001	0.001	0.001						
	R	0.001	0.001	0.001	0.001					
	V	0.001	0.001	0.001	0.001	0.070				
Fd	H	0.001	0.001	0.001	0.001	0.001	0.001			
	R	0.001	0.001	0.001	0.001	0.001	0.001	0.001		
	V	0.001	0.001	0.001	0.001	0.001	0.001	0.001	0.029	

Supplementary Figure S1. Adjusted *p*-values (composition, structure) of pairwise comparisons from the significant interaction term (Species:Tissue) in tests of significant differences among bacterial communities of natural congeners. Shaded values are not significant ($p > 0.05$). Fs = *Fucus spiralis*, Fv = *Fucus vesiculosus*, and Fd = *Fucus distichus*.
