# Supplementary material for: Bacterial Communities Show Algal Host (Fucus spp.)/Zone Differentiation Across the Stress Gradient of the Intertidal Zone
Source: Front Microbiol. 2020 Sep 24;11:563118. doi: 10.3389/fmicb.2020.563118 (PMC7541829; doi:10.3389/fmicb.2020.563118)
Supplement: Supplementary file 2 [file Image_2.pdf]

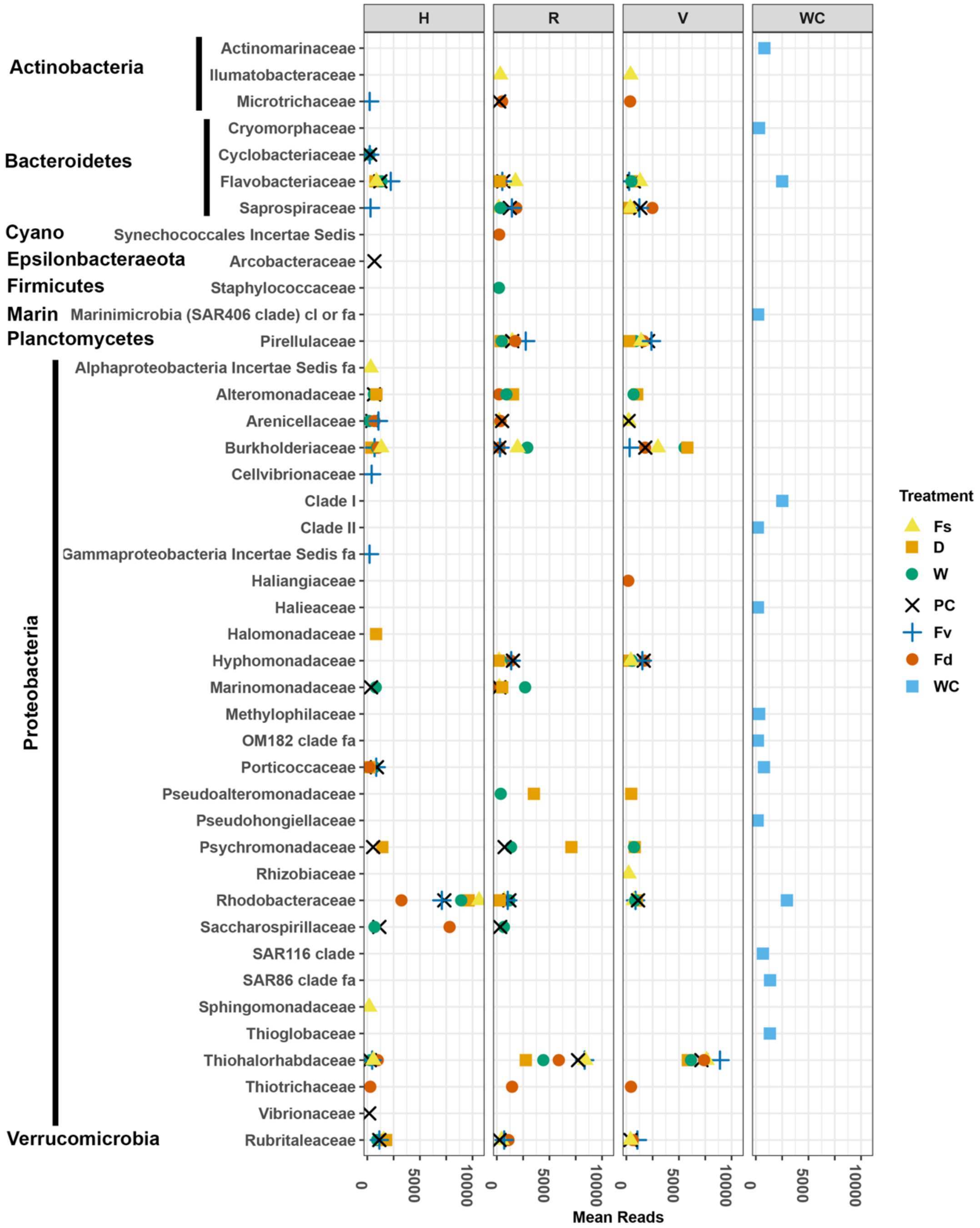

Supplementary Figure S2. Scatter plot of mean reads of major families of bacteria across replicates. Fs = *Fucus spiralis*, D = dry transplant of *Fucus vesiculosus*, W = watered transplant of *Fucus vesiculosus*, PC = procedural control of *Fucus vesiculosus*, Fv = natural *Fucus vesiculosus*, Fd = *Fucus distichus*, WC = water column; H = holdfast, R = receptacle, and V = vegetative. Phyla are shown in bold: Cyano = Cyanobacteria and Marin = Marinimicrobia (SAR406 clade).
