# Supplementary material for: Bacterial Communities Show Algal Host (Fucus spp.)/Zone Differentiation Across the Stress Gradient of the Intertidal Zone
Source: Front Microbiol. 2020 Sep 24;11:563118. doi: 10.3389/fmicb.2020.563118 (PMC7541829; doi:10.3389/fmicb.2020.563118)
Supplement: Supplementary file 3 [file Image_3.pdf]

**Composition**

| | | 6-Jul D | 6-Jul W | 6-Jul PC | 11-Jul D | 11-Jul W | 11-Jul PC | 20-Jul D | 20-Jul W | 20-Jul PC |
|---|---|---|---|---|---|---|---|---|---|---|
| 6-Jul | D | | | | | | | | | |
| | W | 0.452 | | | | | | | | |
| | PC | 0.414 | 0.452 | | | | | | | |
| 11-Jul | D | 0.308 | 0.414 | 0.271 | | | | | | |
| | W | 0.129 | 0.216 | 0.116 | 0.452 | | | | | |
| | PC | 0.321 | 0.414 | 0.363 | 0.452 | 0.414 | | | | |
| 20-Jul | D | 0.069 | 0.069 | 0.041 | 0.069 | 0.069 | 0.069 | | | |
| | W | 0.116 | 0.116 | 0.069 | 0.116 | 0.116 | 0.069 | 0.414 | | |
| | PC | 0.321 | 0.414 | 0.414 | 0.414 | 0.312 | 0.414 | 0.069 | 0.084 | |

**Structure**

| | | 6-Jul D | 6-Jul W | 6-Jul PC | 11-Jul D | 11-Jul W | 11-Jul PC | 20-Jul D | 20-Jul W | 20-Jul PC |
|---|---|---|---|---|---|---|---|---|---|---|
| 6-Jul | D | | | | | | | | | |
| | W | 0.372 | | | | | | | | |
| | PC | 0.362 | 0.372 | | | | | | | |
| 11-Jul | D | 0.177 | 0.362 | 0.147 | | | | | | |
| | W | 0.09 | 0.206 | 0.085 | 0.372 | | | | | |
| | PC | 0.211 | 0.362 | 0.206 | 0.372 | 0.362 | | | | |
| 20-Jul | D | 0.085 | 0.085 | 0.051 | 0.085 | 0.085 | 0.085 | | | |
| | W | 0.109 | 0.147 | 0.085 | 0.089 | 0.147 | 0.089 | 0.362 | | |
| | PC | 0.206 | 0.362 | 0.3 | 0.362 | 0.206 | 0.362 | 0.078 | 0.102 | |

**Supplementary Figure S3.** Adjusted *p*-values (composition, structure) of pairwise comparisons from the significant interaction term (Day:Treatment) for vegetative tissue in the three transplant treatments (dry, watered, procedural control). Shaded values are not significant ($p > 0.05$).
