# Supplementary material for: Bacterial Communities Show Algal Host (Fucus spp.)/Zone Differentiation Across the Stress Gradient of the Intertidal Zone
Source: Front Microbiol. 2020 Sep 24;11:563118. doi: 10.3389/fmicb.2020.563118 (PMC7541829; doi:10.3389/fmicb.2020.563118)
Supplement: Supplementary file 4 [file Image_4.pdf]

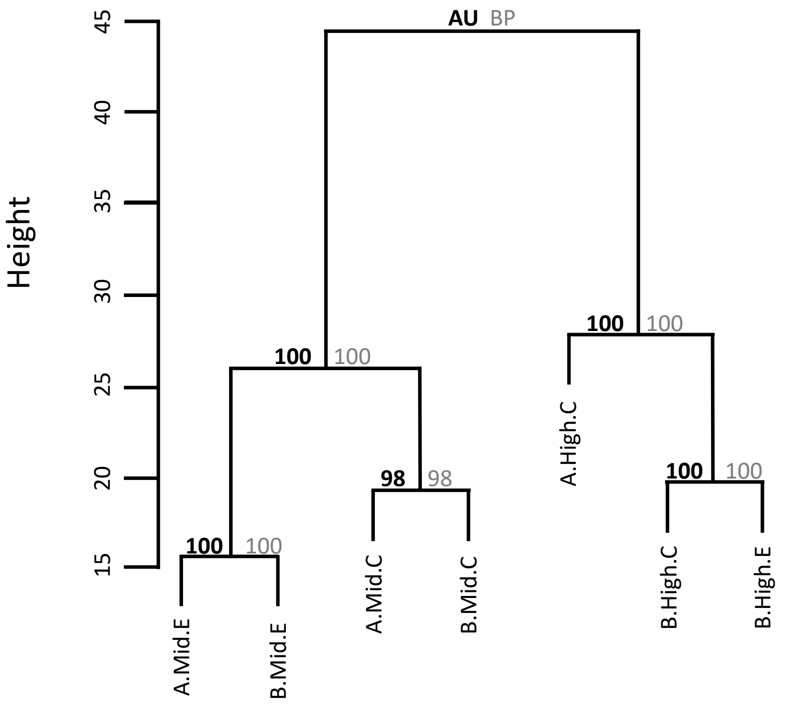

Supplementary Figure S4. Neighbor-joining tree generated from a Euclidean dissimilarity matrix using iButton temperature records. Each branch represents a single record and gives approximately unbiased (AU) p-values (bold) and bootstrap probabilities (BP, in gray).
